# Draft Genome Sequence of Pseudomonas brassicacearum Strain UTMN3, a Biological Control Agent from the Rhizosphere of Pisum sativum

**DOI:** 10.1128/MRA.00895-21

**Published:** 2021-11-11

**Authors:** D. V. Poshvina, A. V. Vasilchenko, A. S. Vasilchenko

**Affiliations:** a Laboratory of Antimicrobial Resistance, Institute of Environmental and Agricultural Biology (X-Bio), Tyumen State University, Tyumen, Russian Federation; b All-Russian Institute of Plant Protection, St. Petersburg-Pushkin, Russian Federation; University of Arizona

## Abstract

Here, we announce and describe the draft genome sequence of Pseudomonas brassicacearum UTMN3, which contains 40 contigs comprising 6,658,810 bp, with a GC content of 60.9%. The genome contains 5,825 protein-coding genes and 65 RNA-coding genes. The genome of UTMN3 contains several genes that are likely contributors to plant protection.

## ANNOUNCEMENT

Pseudomonas brassicacearum strains are ubiquitous soil microorganisms that have long been studied for their beneficial plant growth-promoting and biocontrol properties (1). Members of this group have great potential in biocontrol because of their ability to produce secondary metabolites relevant in the suppression of pathogens (2).

P. brassicacearum strain UTMN3 was isolated from the rhizosphere of a pea plant grown in an agricultural field (Tyumen region, Russian Federation). The strain was isolated and preliminarily identified to the genus level using routine bacteriological methods (3), including biochemical differential strips (Rapid ID 32 E strips; bioMérieux SA, France) (4). The isolated strain was grown in Luria-Bertani (LB) broth at 27°C under aerobic conditions.

Genomic DNA was isolated from an overnight broth culture using a routine method, which included mechanical homogenization followed by enzymatic lysis. Briefly, 300 μl of Tris-salt buffer (100 mM Tris-HCl, 20 mM EDTA, 750 mM NaCl [pH 8.0]) was added to 50 μl sediment of the studied culture and homogenized using a TissueLyser LT instrument (Qiagen, Germany) with lysing matrix E (MP Biomedicals, USA) for 1 min at a frequency of 50 Hz. Subsequently, a 10% SDS solution was added to the mixture to a final concentration of 1% with 4 μl of proteinase K solution (10 mg/ml), and the mixture was incubated for 30 min at 60°C. After extraction with phenol-chloroform-isoamyl alcohol (25:24:1) and subsequent extraction with chloroform-isoamyl alcohol (24:1), DNA was precipitated from the aqueous phase in 3 volumes of absolute ethanol with the addition of 10 M ammonium acetate (1:10) at –20°C overnight. After centrifugation and double washing with 70% ethanol, the DNA was dried and dissolved in 30 μl of deionized autoclaved water. The DNA concentration was estimated using a Qubit 4.0 fluorimeter (Thermo Fisher Scientific, Germany). DNA quality was evaluated using a NanoDrop 8000 spectrophotometer (Thermo Fisher Scientific).

A DNA library was constructed using the NEBNext Ultra II FS library preparation kit (New England Biolabs, USA) according to the manufacturer’s protocols. The target size of the sequenced DNA fragment was 550 bp. Sequencing was performed with a high-throughput MiSeq platform (Illumina, USA) in the Persistence of Microorganisms Center of Shared Scientific Equipment of the Institute of Cellular and Intracellular Symbiosis of the Ural Branch of the Russian Academy of Sciences, using a MiSeq reagent kit v3 with 2 × 300-bp reads.

Sequencing on the Illumina MiSeq platform yielded 3,233,635 paired-end reads. Genome assembly was performed using Unicycler v0.4.9b (5), including assembly using the SPAdes v3.13.1 genomic collector and polishing of the resulting contigs using Pilon v1.23. The resulting assembly contigs were analyzed using QUAST v5.0.2 (6) to assess assembly quality. The prediction of genes encoding rRNA was performed using the barrnap program v0.9. Automatic annotation was conducted with the rapid prokaryotic genome annotation software (Prokka v1.14.6) (7) and the antiSMASH v6 server (8). All mentioned software was used with default parameters unless otherwise specified.

The genome of UTMN3 consists of 6,658,810 bp with 99.2-fold overall coverage and an average GC content of 60.9%. The contig *N*_50_ value was 288,601 bp, and the longest contig was 863,845 bp. Among the 5,957 predicted genes, 5,825 were identified as protein-coding genes. The genome annotation contained 57 tRNAs, 3 rRNAs (5S, 16S, and 23S), and 4 noncoding RNAs.

Genome annotation revealed a number of biosynthetic gene clusters (BGCs). Among them, the BGC for 2,4-diacetylphloroglucinol production is entirely similar to the BGC of Pseudomonas fluorescens F113 (GenBank accession no. AF497760.1), which contributes to phytopathogen suppression.

### Data availability.

The assembled genome sequence of P. brassicacearum strain UTMN3 was deposited in GenBank under accession no. JAFELJ000000000.1. The Illumina MiSeq raw reads were deposited in the NCBI Sequence Read Archive (SRA) under accession no. SRR16318548 (BioProject no. PRJNA700469 and BioSample no. SAMN17832166).
